# Systematic review of statistically-derived models of immunological response in HIV-infected adults on antiretroviral therapy in Sub-Saharan Africa

**DOI:** 10.1371/journal.pone.0171658

**Published:** 2017-02-15

**Authors:** Joseph B. Sempa, Eva L. Ujeneza, Martin Nieuwoudt

**Affiliations:** South African Department of Science and Technology/National Research Foundation Centre of Excellence in Epidemiological Modelling and Analysis (SACEMA), Stellenbosch University, Stellenbosch, South Africa; Azienda Ospedaliera Universitaria di Perugia, ITALY

## Abstract

**Introduction:**

In Sub-Saharan African (SSA) resource limited settings, Cluster of Differentiation 4 (CD4) counts continue to be used for clinical decision making in antiretroviral therapy (ART). Here, HIV-infected people often remain with CD4 counts <350 cells/μL even after 5 years of viral load suppression. Ongoing immunological monitoring is necessary. Due to varying statistical modeling methods comparing immune response to ART across different cohorts is difficult. We systematically review such models and detail the similarities, differences and problems.

**Methods:**

‘Preferred Reporting Items for Systematic Review and Meta-Analyses’ guidelines were used. Only studies of immune-response after ART initiation from SSA in adults were included. Data was extracted from each study and tabulated. Outcomes were categorized into 3 groups: ‘slope’, ‘survival’, and ‘asymptote’ models. Wordclouds were drawn wherein the frequency of variables occurring in the reviewed models is indicated by their size and color.

**Results:**

69 covariates were identified in the final models of 35 studies. Effect sizes of covariates were not directly quantitatively comparable in view of the combination of differing variables and scale transformation methods across models. Wordclouds enabled the identification of qualitative and semi-quantitative covariate sets for each outcome category. Comparison across categories identified sex, baseline age, baseline log viral load, baseline CD4, ART initiation regimen and ART duration as a minimal consensus set.

**Conclusion:**

Most models were different with respect to covariates included, variable transformations and scales, model assumptions, modelling strategies and reporting methods, even for the same outcomes. To enable comparison across cohorts, statistical models would benefit from the application of more uniform modelling techniques. Historic efforts have produced results that are anecdotal to individual cohorts only. This study was able to define ‘prior’ knowledge in the Bayesian sense. Such information has value for prospective modelling efforts.

## Introduction

The successful roll out of antiretroviral therapy (ART) in Sub-Saharan Africa (SSA) has dramatically improved the survival of Human Immunodeficiency Virus (HIV) infected people in this region, which remains a focal point of the HIV epidemic [1]. In the majority of cases, the successful suppression of plasma viral load (VL) after ART initiation to below detection levels facilitates immunological recovery in the form of rising CD4 (+) T cell counts. However, ‘residual viremia’, involving the multiplication of the virus, within for example gut reservoirs, may continue even after circulating VL has been suppressed [2]. As a result CD4 cell count depletion may continue in long term treatment [2,3]. Patients particularly at risk of secondary opportunistic infections include immune ‘non-responders’ who have low CD4 counts in spite of a suppressed VL [2].

In resource limited settings (RLS) such as SSA, CD4 counts continue to be used for clinical decision making, e.g. when to initiate first-line, switch to second-line ART [4] and to benchmark the risk of incident clinical events [5,6]. In this region, patients who fail to reach >350 cells/μL after 5 years of ART [7] are common and ongoing immunological monitoring is necessary. CD4 count is more affordable than VL monitoring and continues to be the only immunological biomarker recommended by the World Health Organization [8].

However, as a biomarker, CD4 counts are known to be inherently variable both within and between individuals [2,9]. Further, prior multivariate models of CD4 count response to ART have employed varying outcome measures and have consequently produced inconsistent results [10–17]. This variation in models complicates the effects of inherent variation in CD4 counts and hinders the comparison of immunological responses to ART across different cohorts.

In this study we systematically review statistical, or empirically-derived rather than biological-mechanistic mathematical, models of immunological response (CD4 counts) in SSA cohorts. We highlight the similarities, differences and problems associated with the varying methodologies with the aim of defining prior knowledge, in the Bayesian sense, for prospective modeling exercises in the future.

## Methods

### Search strategy

The guidelines from the Preferred Reporting Items for Systematic Review and Meta-Analyses (PRISMA) were used (S1 File) [18]. The search syntax was constructed around 4 major terms, allowing for small variations within each. These included ‘immune response’, ‘HIV antiretroviral treatment’ or ‘ART’, ‘Statistical model’, and ‘Sub Saharan Africa’ or ‘SSA’. Each term was defined based on Medical Subject Heading (MESH) terms or other common, published terminology. Online electronic databases were searched using SCOPUS [19], from 1^st^ January 2004 up to 2^nd^ April 2015 (S2 File). This start date was selected as it corresponds to the commencement of ART scale-up in most of SSA [4]. Only studies published in peer-reviewed English-language journals, which existed in all four sets mentioned above were selected.

### Study selection

Abstracts and full-texts of potentially relevant studies were reviewed by JBS and ELU. MN provided the deciding vote if consensus was not unanimous regarding the inclusion or exclusion of a study. Only studies of immunological response, measured as an outcome in any form, after ART initiation in adults were included. Although immune response is not limited to CD4, our searches only returned modeling studies that employed it. Studies were excluded where: 1. there was no multivariate statistical model, 2. immune response was combined with any other treatment outcome, 3. data was analyzed that contained a combination of people from SSA with those from other regions, and 4. immune response prior to ART initiation was analyzed.

Model outcomes were categorized into 3 general groups, further sub-divided by the type of regression used:

1. the trajectory of CD4 counts within particular time-frames after ART initiation, or ‘slope’ models, with Generalized Estimating Equations (GEE), and Generalized Linear Mixed Effects (GLME),
2. the time to a particular immune response, or ‘survival’ models, with Cox Proportional Hazards (CPH) and
3. the specified overall gain in CD4 count, or ‘asymptote’ models, with Logistic, Simple Linear, Difference-in-Difference, Log-Binomial and Poisson regression.

### Data extraction

The following data was extracted from each study: first author, year published, country, the sex/es studied, sample size, study design, ART follow-up years, initiating ART regimen (if reported), outcome/s analyzed, variable scale transformation methods, criteria for model variable/s selection (e.g. statistical methods and/or *a priori* clinical information), assessment of confounding and covariates adjusted for in the final model. For each of the final model variables, the unit and scale of measurement, effect sizes, 95% confidence intervals and, where available, standard deviations were noted. Effect sizes were rounded off to the nearest whole number and 95% confidence intervals and standard deviation to one decimal place. If ‘immunologic failure’ was mentioned, we checked if it was defined according to the WHO criteria [4].

Risk of bias was also assessed in each study as follows: Low risk—covariate adjusted for in model based on its clinical/biological plausibility; medium risk—covariates included based on both biological and statistical significance; and high risk—model employed only statistical significance (p-value). The provision by authors of biological reasoning, including references, for their covariate adjustments was noted.

### Statistical analysis

All data was collated in MS Excel (version 2013) and comparisons made as per the tables below. In R version 3.2.2 using package ‘wordcloud’ [20], variables adjusted for in the final multivariate models were presented. In wordclouds, the size and color of each word is determined by the frequency of its appearance in a list, in this case all covariates adjusted-for within a specified outcome. This enabled the comparison of variables with potentially different units and/or numeric scales. A minimal frequency cutoff of ≥3 was used to define the ‘consensus’ set of covariates across all models reviewed.

## Results

**Fig 1:**
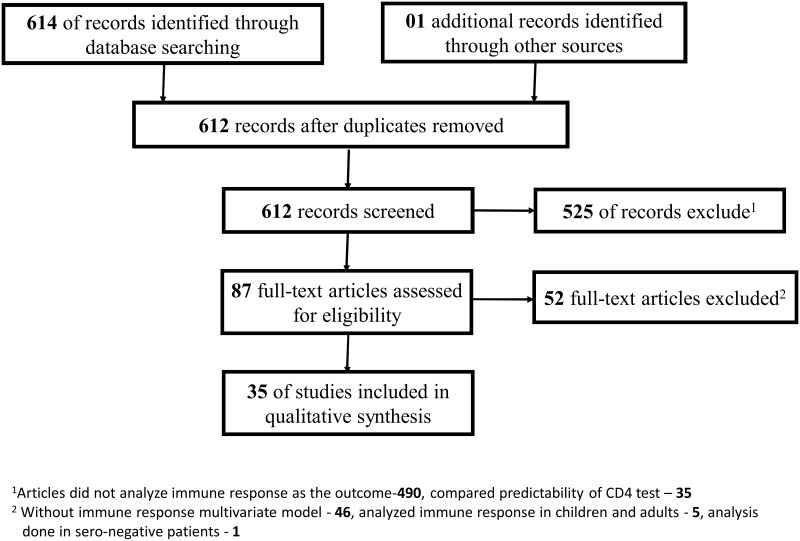
Systematic review flow chart.

**Fig 2:**
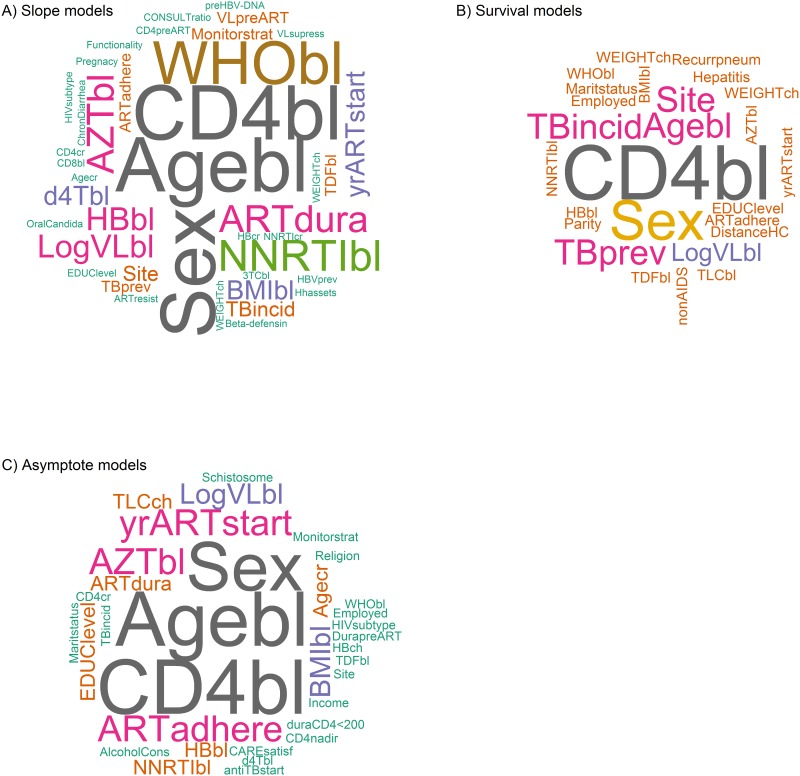
Wordclouds for the categorized immune response outcomes from SSA models. *Figure 2A*: Covariates adjusted for in the final slope models; *Figure 2B*: Covariates adjusted for in the final Survival models; and *figure 2C*: Covariates adjusted for in the final Asymptote models. The word size and color represents the frequency of covariates, hence the larger the size of the covariate, the higher its frequency in the list of adjusted covariates. **Site**—location of the study; **KSincid**—Kaposis’ sarcoma diagnosed after ART start; **HBVprev**—Hepatitis B virus diagnosed at ART start; TBprev—History of TB at ART start; **TDFbl**—treated with tenofovir at ART start; **3TCbl**—treated with lamivudine at ART start; **DistanceHC**—distance from health center; **Maritstatus**—marital status of the subject; **Season**—season of the tear when patient was initiated on ART; **ALTbl**—alanine aminotransferase at ART start; **sdNVP**—history of single does nevirapine; **Parity**—number of children; **CD8bl**—CD8 count at ART start; **CONSULTratio**—cadre levels at health center; **Hhassets**—possession of any household assets; **OralCandida**—Oral candidiasis at ART start; **ChronDiarrhea**—Chronic diarrhea at ART start; **VLsupress**—ever had viral suppression; **NNRTIcr**—time-updated exposure to either nevirapine or efavirenz; **NRTI**_**cr**_—time-updated exposure to **d4T**_**cr**_ (stavudine) or **AZT**_**cr**_ (zidovudine) or **TDF**_**cr**_ (tenofovir) or **3TC**_**cr**_ (lamivudine); **CD4preART**—pre-ART start CD4 count; **VLpreART**—pre-ART start viral load; **PreARTexp**—pre-ART exposure; **AlcoholCons**—consumption of alcohol; **DurapreART**—duration between ART start and diagnosis; **duraCD4<200**—duration while CD4 <200 cells/μL before ART start; and **antiTBstart**—patient initiated on anti-tuberculosis medicine. *For other variable definitions, please refer to the notes below* Tables 2, 3 and 4.

Of the 615 articles identified 580 were excluded based on the specified inclusion criteria (Fig 1). Of the remaining 35 the median sample size (and IQR) was 1002 (351–5448) with follow-up of 2 years (1–5). Across all models, 75 unique covariates were included in multivariate analysis, of which 69 were adjusted for in the final models. In the majority of cases the effect sizes of covariates were not directly comparable in view of the combination of different variables and varying scale transformations methods across models. However, the frequency of the occurrence of variables, independent of their scales, enabled the identification of a consensus set (Fig 2).

For slope models this included, gender, baseline age, baseline CD4 count, baseline WHO stage, ART initiating or ‘baseline’ regimen, e.g. efavirenze vs nevirapine, baseline exposure to zidovudine or stavudine, ART duration, log-VL, baseline hemoglobin level, baseline Body Mass Index (BMI), year of ART start, study site and tuberculosis incidence. For survival models, baseline CD4 count, gender, baseline age and either prevalent or incident tuberculosis. For asymptote models, gender, baseline age, baseline CD4 count, baseline zidovudine exposure, year of ART start, ART adherence, log-VL and baseline BMI. Across all three types of models, Sex, Age, baseline log VL, baseline CD4, ART initiation regimen and ART duration count were the most commonly adjusted for covariates and also those most often significantly associated with the immunological outcomes (Table 1).

**Table 1:** The high frequency (≥3) covariates adjusted for in multivariate models.

Description	Slope models	Survival models	Asymptote models
**Baseline CD4 count**	13	7	9
**Sex of the participants**	13	5	8
**Age at baseline**	13	3	9
**WHO stage at baseline**	10	1	1
**Type non-nucleoside reverse transcriptase Inhibitor (i.e efavirenze or nevirapine)**	7	1	2
**Initiated on zidovudine at baseline**	6	1	4
**Duration while on antiretroviral therapy**	6	0	2
**Log_10_ viral load at baseline**	5	2	3
**hemoglobin level at baseline**	5	1	2
**Calendar year of ART start**	4	1	4
**Body Mass Index at baseline**	4	1	3
**Initiated on stavudine at baseline**	4	0	1
**Location of treatment program or clinic**	3	3	1
**Incident tuberculosis diagnosis after ART start**	3	3	1
**History of TB at baseline**	2	3	0
**Antiretroviral therapy adherence**	2	1	4

Notes:

‘Baseline’—Refers to the measurement at ART initiation

Differences were found in the estimation of effect sizes and residuals across all 19 slope models (Table 2, below). Two authors reported using GEEs without additional details [13,21]. Hermans et al. 2010 used a GEE with robust standard errors and exchangeable correlation matrix [16]. Hawkins et al. 2011 applied GEE with step-wise restricted cubic splines to fit the non-linear CD4 count response [22]. Sudfeld et al. 2012 and Sudfeld et al. 2013 used GEE with restricted cubic splines and an m-dependent correlation matrix [23,24]. Hardwick et al. modeled slope of CD4 count using a GEE model with type 3 sums of squares and variance correction to correct for longitudinal CD4 count time points [25]. Boullé et al. 2013, Velen et al. 2013, Schomaker et al. 2013, Hamers et al. 2012, and Hamers et al. 2013 used GLMEs of slope of CD4 count [26–30]. Maman et al. 2012 and Reda et al. 2013 used GLME with random intercept and coefficients, while the former extended this by adding a second degree polynomial for time on ART [31,32]. Maskew et al. 2013 used GLME with random slope and intercepts and specified an unstructured correlation matrix for repeated measures [33]. Mayanja et al. 2012 and Wandeler et al. 2013 used a GLME with functional polynomials [14,34]. Sarfo et al. 2014 used GLME with a log-link and assumed a Poisson distribution for CD4 count response [35], while De Beaudrap et al. 2009 applied a non-linear mixed effects model [36]. Vinikor et al 2014 used analysis of covariance (ANCOVA) [37].

**Table 2:** ‘Slope’ models of CD4 count trajectory in SSA.

Authors	Location	Period	Study size	End point	Significant covariates
**Hermans et al. 2010** [16]	Uganda	2003–09	5982	Mean CD4 count change from baseline	TBincid, CD4_bl_, sex
**Peterson et al. 2011** [21]	The Gambia	2004–09	359	Mean CD4 count change from baseline	LogVL_bl_, CD4_bl_, ART_dura_
**Hawkins et al. 2011** [22]	Tanzania	2004–08	12842	Mean CD4 count change between visits	Sex
**Mayanja et al. 2012** [34]	Uganda	2004–09	88	Mean CD4 count response	ART_dura_, Pregnancy and their interaction, CD4preg, TIMEpreg
**Sudfeld et al. 2012** [24]	Tanzania	2006–10	875	Mean CD4 count change between visits	None reported
**Hardwick et al. 2012** [25]	Ethiopia and Tanzania	No details	1002	Mean CD4 count response	Beta-defensin
**Maman et al. 2012** [31]	Malawi, Uganda, Kenya	2001–09	12946	Mean CD4 count response	Sex, site, Age_cr_, CD4_bl_
**Maskew et al. 2013** [33]	South Africa	2008–09	232	Mean CD4 count change from baseline	Sex, CD4_bl_, Age_curr_
**Sempa et al. 2013** [13]	Uganda	2004–12	356	Mean CD4 count change from baseline	Sex, CD4_bl_, log VL_bl_, AZT_bl_, ART_t_, HB_cr_
**Boullé et al. 2013** [26]	Cameroon	2006–10	459	Mean CD4 count response	Sex, Age_bl_, logVL_bl_, ART_dura_
**Reda et al. 2013** [32]	Ethiopia	2005–10	1540	Mean CD4 count response	ART_dura_
**Sudfeld et al. 2013** [23]	Tanzania	2006–09	2145	Mean CD4 count change between visits	None reported
**Velen et al. 2013** [27]	South Africa	2007–2009	6196	Mean CD4 count response	d4T_cr_, AZT_cr_, TDF_cr_
**Wandeler et al. 2013** [14]	Southern Africa	No details	72597	Mean CD4 count response	AZT_cr_
**Schomaker et al. 2013** [28]	South Africa	2003–10	15646	Mean CD4 count change between visits*	Sex, TBincid, CD4_bl_, Age_bl_, WHO_st_
**Sarfo et al. 2014** [35]	Ghana	2004–10	3990	Gains in CD4 count	CD4_bl_, Age_bl_, YrARTstart, Sex, WHO_st_, NRTI_bl_, NNRTI_bl_, ART_dura_
**Vinikoor et al. 2014** [37]	Zambia	2004–10	43152	Mean CD4 count change from baseline	Age_bl_
**Hamers et al. 2012** [29]	Kenya, Nigeria, South Africa, Uganda, Zambia, and Zimbabwe	2007–09	2439	Mean CD4 count response	ARTresist
**Hamers et al. 2013** [30]	Zambia and South Africa	2007–08	1127	Mean CD4 count response	None reported
**De Beaudrap et al. 2009** [36]	Senegal	1998–07	346	Mean CD4 count response**	CD4_bl_, and logVL_bl_

*cells/μL per 6 months;

**Square root cells/μL

**Note: site**—study location; **Age**_**bl**_—baseline age; **Age**_**cr**_—current age; **WHO**_**st**_—baseline WHO stage; **Log**_**10**_**VL**_**bl**_—baseline Log Viral Load; **CD4**_**bl**_—baseline CD4count; **HB**_**cr**_—current hemoglobin level; **YrARTstart**—year of ART start; **ART**_**dura**_—duration on ART; **AZT**_**bl**_ exposure to zidovudine at baseline; **NRTI**_**bl**_—exposure to **d4T**_**bl**_ (stavudine) or **3TC**_**bl**_ (lamivudine) at ART start; **NNRTI**_**bl**_—exposure to either efavirenze or nevirapine at ART start; **ARTresist**—pre-ART drug resistance; **TBincid**—Incident tuberculosis diagnosis after ART start; **TIMEpreg**—duration between pregnancies; **CD4preg**—whether CD4 count was taken during pregnancy;

More similar methods were employed in Survival and Asymptote models to estimate effect sizes and their residuals. In survival models, six out of seven models estimated effect sizes using CPHs [17,22,38–40,42] and Teshome et al. 2014 used a stratified-CPH [41] (Table 3). Four variants of asymptote models, were found: 1. Factors associated with reaching a particular threshold CD4 count or not, analyzed using multivariate logistic regression [14,16,37,43–46], 2. Overall change in CD4 count, using multivariate linear regression [15,21,47,48], 3. Maskew et al. 2013 assumed the outcome followed a log-binomial distribution [49]; and 4. Takuva et al. 2012 assumed a Poisson distributed with robust standard errors [50], see Table 4.

**Table 3:** ‘Survival’, or time-to immune response, models in SSA.

Authors	Location	Period	sample size	End point * ^(criteria)^	Significant covariates
**Assefa et al. 2014** [17]	Ethiopia	2007–11	400	Time to immunologic failure ^α^^, (^^4^^)^	Sex, CD4_bl_
**Kigozi et al. 2009** [38]	Uganda	2002–06	427	Time to CD4 increase ≥50 cells/μL	nonAIDS, CD4_bl_, ARTadhere, TLC_bl_
**Palladino et al. 2013** [39]	Mozambique	2002–06	142	Time to immunologic failure ^α^^, (^^4^^)^	CD4_bl_, Log_10_VL_bl_
**Alemu Melsew et al. 2013** [40]	Ethiopia	2007–12	509	Time to immunologic failure ^α^^, (^^4^^)^	Recurrpneum, Employed, WEIGHT_ch_, CD4_bl_
**Teshome et al. 2014** [41]*	Ethiopia	2004–12	268	Attain ^α^^, (^^4^^)^	CD4_bl_
**Hawkins et al. 2011** [22]	Tanzania	2004–08	762	Time to immunologic failure ^α^^, (^^1^^,^ ^2^^,^ ^3^^, &^ ^4^^)^	Sex
**Mudiope et al. 2013** [42]	Uganda	2003–11	289	Time to immunologic failure ^α^^, (^^4^^)^	CD4_bl_

* Case-control study;

^α^ WHO criteria;

^1^ CD4 cell count falls below baseline in the absence of other concurrent infections

^2^ CD4 cell count falls to less than 50% of peak levels without coexistent infections

^3^ CD4 cell count is persistently below 100 cells/μL

^4^ Any one of the 3 criteria above

**Note: Log**_**10**_**VL**_**bl**_—baseline Log Viral Load; **CD4**_**bl**_—baseline CD4count; **TLC**_**bl**_—baseline total lymphocyte count; **ARTadhere**—Antiretroviral therapy adherence; **WEIGHT**_**ch**_—change in weight from baseline; **Recurrpneum**—recurrent pneumonia; **Employed**—employment status; **nonAIDS**—AIDS or non-AIDS defining conditions

**Table 4:** ‘Asymptote’ models in SSA.

Authors	Location	Period	sample size	End point	Significant covariates
**Anude et al. 2013** [43]	Nigeria	2008–09	596	CD4 count increase ≥50 cells/μL	Sex, Age_bl_,
**Efraim et al. 2013** [46]	Tanzania	2009–11	351	Attain ^α^^, (^^1^^,^ ^2^^)^	Schistosome, BMI_bl_, CD4_bl_, EDUClevel
**Hermans et al. 2010** [16]	Uganda	2003–09	5982	Attain ^α^^, (^^3^^)^	TBincid, CD4_bl_, AZT_bl_
**Diabaté et al. 2009** [44]	Ivory coast	2005	303	CD4 count increase ≥50 cells/μL	ARTadhere, TLC_ch_
**Wandeler et al. 2013** [14]	South Africa, Botswana, Zambia, and Lesotho	No details	14529	Attain ^α^^, (^^3^^)^	AZT_cr_, sex, Age_bl_, CD4_bl_, HB_bl_, YrARTstart, Monitorstrat
**Maskew et al. 2013** [49]	South Africa	2001–08	8676	CD4 count increase ≥50 cells/μL or ≥100 cells/μL	None reported
**Nglazi et al. 2011** [45]	South Africa	2002–08	3162	CD4 ≤200 cells/μL at week 48	Sex, Age_bl_, CD4_bl_, VL_bl_
**Vinikoor et al. 2014** [37]	Zambia	2004–10	43152	Attain CD4 count ≥350 cells/μL	Age_bl_
**McKinnon et al. 2010** [48]	Kenya	2005–11	60	Overall change in CD4 count	CD4nadir
**Alemu et al. 2012** [47]	Ethiopia	2009–10	1722	Overall change in CD4 count	Depression, SOCIALsup
**Crawford et al. 2015** [15]	Uganda	2011	325	Overall increase in CD4 count	CD4_cr_, ART_dura_, Age_bl_, CAREsatisf, and TLC_ch_ HB_ch_
**Peterson et al. 2011** [21]	The Gambia	2004–09	359	Overall increase in CD4 count	HIVsubtype, ART_dura_, and their interaction
**Takuva et al. 2012** [50]	South Africa	2004–09	1499	CD4 count increase ≥50 cells/μL	None reported

^α^WHO criteria;

^1^.CD4 cell count falls below baseline in the absence of other concurrent infections,

^2^.CD4 cell count is persistently below 100 cells/μL

^3^ Any one of the criteria above

**Note: Age**_**bl**_—baseline age; **BMI**_**bl**_—Body Mass Index; **EDUClevel**—level of education; **CD4**_**bl**_—baseline CD4count; **CD4**_**cr**_—current/most recent CD4 count; **HB**_**bl**_—hemoglobin level at ART start; **HB**_**ch**_—change in hemoglobin; **YrARTstart**—year of ART start; **ART**_**dura**_—duration on ART; **AZT**_**bl**_—exposure to zidovudine at baseline; **AZT**_**cr**_—current exposure to zidovudine; **TBincid**—incident tuberculosis; **TLC**_**ch**_—change in total lymphocyte count; **Monitorstrat**—monitoring strategy (clinical or immunological or virological); **Depression**—symptoms depression while on ART; **SOCIALsup**—perceived social support; **CAREsatisf**—patient satisfaction with care; **CD4nadir**—nadir CD4 count; **HIVsubtype**—HIV-1 subtype

For criteria used to select covariates for final multivariate models and assessment for confounding (Table 5), 9 studies reported using significance cutoffs ranging from 0.05 to 0.25, and biological plausibility, i.e. the causal association between the immune response and the covariate, to generate this list of covariates [13,16,22,24,26,38,41–43]. Three authors used only statistical significance (p-values) as a basis for covariate selection in multivariate analysis [29,30,44]. Four of the above 12 studies employed step-wise regression [16,29,30,43], and two used step-wise regression and ‘prior’ reasoning to arrive at their final multivariate model [23,26]. Mayanja et al. 2012 listed model assumptions based on biological CD4 dynamics [34] and Sudfeld et al 2013 referred to prior studies [23]. Only two studies assessed covariates for confounding [25,33].

**Table 5:** Summary of different multivariate immune response modeling methods in SSA.

Author	Criteria for selecting variables into the multivariate model			How they arrived at the Final model		Confounding
	Biological plausibility	Cutoff used	Cutoff	Stepwise selection only	Step-wise and a priori	Assessed confounding
**Anude et al. 2013** [43]	✓	✓	0.20	✓	0	0
**Assefa et al. 2014** [17]	✓	0		0	0	0
**Efraim et al. 2013** [46]	✓	0		✓	0	0
**Hermans et al. 2010** [16]	✓	✓	0.20	✓	0	0
**Kigozi et al. 2009** [38]	✓	✓	0.05	0	0	0
**Maman et al. 2012** [31]	✓	0		0	0	0
**Maskew et al. 2013** [49]	✓	0		0	0	0
**Maskew et al. 2013** [33]	✓	0		0	✓	✓
**McKinnon et al. 2010** [48]	✓	0		✓	0	0
**Palladino et al. 2013** [39]	0	0		0	0	0
**Reda et al. 2013** [32]	✓	0		0	0	0
**Sempa et al. 2013** [13]	✓	✓	0.20	0	0	0
**Sudfeld et al. 2012** [24]	✓	✓	0.20	0	✓	0
**Teshome et al. 2014** [41]	✓	✓	0.05	0	0	0
**Velen et al. 2013** [27]	✓	0		0	0	0
**Alemu Melsew et al. 2013** [40]	0	0		0	0	0
**Alemu et al. 2012** [47]	0	0		0	0	0
**Boullé et al. 2013** [26]	✓	✓	0.25	0	✓	0
**Crawford et al. 2015** [15]	0	0		✓	0	0
**Daibaté et al. 2009** [44]	0	✓	0.25	0	0	0
**Hamers et al. 2012** [29]	0	✓	0.10	✓	0	0
**Hamers et al. 2013** [30]	0	✓	0.15	✓	0	0
**Hardwick et al. 2012** [25]	✓	0		0	✓	✓
**Hawkins et al. 2011** [22]	✓	✓	0.20	0	0	0
**Mayanja et al. 2012** [34]	✓	0		0	0	0
**Mudiope et al. 2013** [42]	✓	✓	0.20	0	0	0
**Peterson et al. 2011** [21]	✓	0		0	0	0
**Sarfo et al. 2014** [35]	✓	0		0	0	0
**Sudfeld et al. 2013** [23]	✓	0		0	0	0
**Vinikoor et al. 2014** [37]	✓	0		0	0	0
**Wandeler et al. 2013** [14]	✓	0		0	0	0
**Nglazi et al. 2011** [45]	✓	0		0	0	0
**Takuva et al. 2012** [50]	0	0		0	0	0
**Schomaker et al. 2013** [28]	✓	0		0	0	0
**De Beaudrap et al. 2009** [36]	✓	0		✓	0	0

## Discussion

This study systematically reviewed recent statistical or empirically-defined models of CD4 count response in HIV-infected adults on ART in SSA. The aim was to arrive at a set of model covariates and outcomes that might allow the comparison of modeling results between cohorts. From the studies reviewed, Sex, Age, baseline log VL, baseline CD4, ART initiation regimen and ART duration were the most commonly adjusted covariates and also those most often significantly associated with the different metrics of immune response across all models reviewed. Many permutations were found, in fact, the majority of the models were different with respect to variable transformations and scales, varying model assumptions, modeling strategies, model reporting methods and the use of different covariates, even if the same outcomes had been studied. In particular:

In the CPH models studied, authors did not adjust for time-updated variables [38–40,42]. It was assumed that patients remained on their initiation regimen throughout the period of follow-up. It is known from studies of ART regimen durability and tolerability that drug toxicity will often occur in the period soon after initiation, necessitating drug substitutions [51,52]. Such switches are obviously important in understanding CD4 responses, particularly if more potent drugs are subsequently employed. ‘Joint’ time-to-event and longitudinal (or repeated) measure models may be used for time-updated covariates, in which a 2-phase process involves combining the model/s of the endogenous longitudinal covariate/s with a CPH model [53].

All seven studies which analyzed time to immunological failure did so for only the time to the first failure episode [17,38–41]. However, it has been conjectured that multiple failures may actually occur and be hidden by the normal variability seen in adult CD4 counts [9]. CPH models are not appropriate for multiple failure-time points since the outcome terminates after the first event. Further, the assumption of the independence of outcomes is violated since events within an individual are correlated [54]. Corrections to such models for correlated failure time points have been implemented in the form of Andersen-Gill, Marginal Wei-Lin-Weissfeld or Prentice-Williams-Peterson methods [54,55]. If multiple episodes of immunologic failure are present, as defined by the WHO criteria, then the Andersen-Gill method would appear to be a good choice [55].

In selecting regression methods, considerations regarding covariate distributions and the mathematical assumptions regarding their relationship/s with the outcome are important. These assumptions can be tested *a priori* using the dataset at hand. Only 4 of the studies reviewed indicated that such tests had been used to confirm that the particular covariates fulfilled the model assumptions [15,23,32,41]. If the assumptions are violated it is not possible to estimate the effect of the covariates on the outcome with both precision and accuracy [56].

Six studies used GEEs to model the slope of the CD4 count response. Three defined the outcome as the change in CD4 count from baseline, i.e. from ART initiation [13,16,21] and the others used the change in CD4 count between each subsequent visit [22–24]. Of the 11 studies that used GLMEs, 2 used the increase from the baseline as outcome [32,33], one used change in CD4 count between subsequent visits [27] and 8 used absolute change in CD4 counts over time [14,25,26,28–31,34]. Only one study used non-linear mixed effects regression [36]. Selecting either GEE—population averaged effects, or GLMM—individual averaged effects, is possible using tests of assumptions regarding the underlying mechanisms of CD4 count response [57]. CD4 counts vary due to both individual patient characteristics and laboratory procedures [58–60]. Given the individual effects, GLMEs may be preferable to GEEs in this context. Non-Linear Mixed Effect models (NLMEs) may also be used since they take into consideration mechanistic biological assumptions and both the underlying subject-specific longitudinal responses (CD4) and the variation of these across the study group over time [57].

Sarfo et al 2014 [35] modelled CD4 count response using a GLMM with a Poisson distribution. Baseline CD4 counts, being female, increasing ART duration and baseline WHO stage (stage 1 and stage 2) where associated with increasing CD4 counts, while initiating ART on efavirenz and zidovudine based regimens and higher baseline age were associated with decreasing CD4 counts. These results apparently support prior studies [2,61]. However, the incident rate ratios (IRR) in their final model were close to null. The Poisson distribution assumption may have biased the results towards the null and presumably explains their rounding off IRR to 3 decimal places of [35]. The Poisson model assumes that the probability of the occurrence of any two events *p*(*x*∩*y*) is negligible, and the probability of the occurrence of an event *p*(*x*) is constant throughout the interval, Δ*t*. In [35] the sampling frequency for CD4 count was 6 monthly, thus, the probability of having another CD4 count measurement was never negligible. Further, the probability of increasing CD4 counts throughout the sampling interval is variable due to adherence, opportunistic infections, and drug resistance [62].

There was also variation in approaches to adjustment for confounding between covariates. Confounding usually refers to a ≥10% change in the coefficient estimate of the main predictor after adjusting for the effect of a covariate [56]. It does not relate to the significance of the p-values for covariates in the model. Four studies did not report the criteria used to select covariates to be adjusted for in the multivariate models [39,40,47,50]. In others [36,38,41,46], covariates were excluded from the final model since they were not statistically significant. This practice may exacerbate confounding [63,64]. Directed Acyclic Graphs (DAGs) can be used as a non-statistical modeling strategy for multivariate analysis [65]. Such causal diagrams, which are based on clinical or biological assumptions, are useful for deciding on the minimal set of covariates to adjust for. Some studies [17,40,42,43,46] did not adjust for covariates, such as age and baseline CD4 count, even if appropriate data had been collected. Prior reviews by Pinzone et al 2012 [2] and Corbeau et al. 2011 [61] have shown that both baseline age and baseline CD4 count are associated with immunological response to ART.

Covariate scale transformations were reported to have been assessed in only five studies [14,24,25,32,34]. Others, report a square root transformation of CD4 counts [14,32,36]. Variable transformations are obviously important in meeting the distributional assumptions of the model/s [56]. Reda [32] investigated a wide range of variable transformations for all variables in their model, while Sudfeld et al 2012 [24] transformed only the main predictor—Vitamin D levels. Other studies employed polynomial transformations of time on treatment [14,25,34] or regression splines on time [22–24,28]. Graphical inspection of the effect of covariate transformation are possible prior to modelling, while statistical tests such as Akaike’s Information Criterion (AIC) and the Bayesian Information Criterion (BIC) are useful afterwards [66]. It is also possible to apply Martingale residuals [55] for CPHs. Caution is always required in variable transformation since, for example, categorizing continuous variables may result in residual confounding [67,68]. Further, the interpretation or translation of results into practice becomes problematic as it is no longer direct.

In terms of model validation, only 5 out of 34 studies provided goodness of fit metrics. These included the AIC [15], Hosmer-Lemeshow test [43], and the Log-likelihood ratio [31,32,45] goodness of fit tests. Other possible techniques include cross-validation, i.e. regressing the model on the training dataset to see if it still predicts the outcome, and graphical methods, i.e. analyzing whether model residuals are random by plotting predicted versus observed values. Without such validations there is a risk of overfitting to data [56]. Similarly, the dissemination of results also has a bearing on the comparability of models. Six studies reported only p-values without beta coefficients or confidence intervals [22–25,46,48] and two studies reported only model coefficients and p-values [34,46]. Ideally, both model coefficients and confidence intervals should be reported. Significant p-values continue to be commonly employed in modeling practice, but these do not indicate clinical significance nor the precision of parameter estimates [69].

Criticism of routine CD4 monitoring in ART has occurred due to the innate biological variation in these counts [9]. However, the value of such criticism seems questionable when it is presented in the absence of suggestions for alternatives, particularly given the fact that HIV is a disease which targets the immune system. Arguably, the limitation of immunological monitoring to only CD4, particularly in SSA, has been based more on considerations of public-health affordability than individual patient welfare. Alternative biomarkers, though considered as indirect immune markers [3], have existed for some time, including among others: Natural Killer (NK) cells, which secrete interferon activating macrophages, which in turn feed off infected and stressed cells and Plasmacytoid Dendritic Cells, which secret type-1 antiviral interferons [3]; β-defensins, which aid in the production of NK cells have also been associated with immunologic response [25,70]; and Co-stimulatory CD28 or co-inhibitory cytotoxic T-lymphocyte antigen 4 proteins, which are expressed by all T-cells in HIV infected people [2,3,71]. The possibility obviously exists to use a combination of CD4 and alternative biomarkers to provide a robust description of the immune system in ART.

This study has limitations. Publication bias may be present in view of the inclusion of only studies published in peer reviewed journals. While specified in the inclusion criteria, only statistical or empirically-derived models were reviewed. This excluded those originating in mechanistic biological theory but did include those expressly incorporating assumptions regarding biological causality. All data collected regarding the models was contingent on the information provided in each study, and based on the assumption that these models should be reproducible using other similar datasets. In comparing the frequency of variables across models, we used a threshold of ≥3 which may have excluded ‘rare’ covariates in SSA cohorts. Only a small number of studies analyzed covariates on comparably transformed or untransformed scales. This negated the possibility of a meta-analysis, i.e. direct quantitative comparisons, since the models adjusted for varying sets of covariates. This situation may be understandable in terms of the facts that certain studies aimed at elucidating particular treatment effects, and that authors are incentivized to publish unique results.

In conclusion, for purposes of comparing immunological, i.e. CD4 count, outcomes across cohorts in SSA, statistical models would benefit from the application of more uniform modelling techniques. The value of the historic models to public health in SSA is questionable since the modeling was apparently performed in the absence of *a priori* comparisons across studies. That is, since such efforts have produced results that are anecdotal to individual cohorts only. However, this study was able to define ‘prior’ knowledge, in the Bayesian sense. Qualitative and semi-quantitative, rather than quantitative and completely comparable effect sizes, for variables in models of immunological response to ART were defined. Such information has value in terms of prospective modeling efforts in the future.

## Supporting information

S1 File PRISMA checklist. (DOCX)

S2 File Search strategy and syntax applied in scopus. (DOCX)

## References

[1] World Health Organization (2015) GLOBAL HEALTH SECTOR RESPONSE TO HIV, 2000–2015: Focus on innovations in Africa. 16 p. http://apps.who.int/iris/bitstream/10665/198148/1/WHO_HIV_2015.40_eng.pdf. Accessed 26 January 2016.

[2] Pinzone MR, Di Rosa M, Cacopardo B, Nunnari G (2012) HIV RNA suppression and immune restoration: can we do better? Clin Dev Immunol 2012: 515962 Available: http://www.pubmedcentral.nih.gov/articlerender.fcgi?artid=3318265&tool=pmcentrez&rendertype=abstract. Accessed 28 May 2014. 10.1155/2012/515962 22489250 PMC3318265

[3] Abbas AK, Lichtman AH, Pillai S (2012) Basic Immunology: Functions and Disorders of the Immune System Basic Immunology: Functions and Disorders of the Immune System. Elsevier USA pp. 303–305.

[4] World Health Organization Joint United Nations Programme on HIV/AIDS (2003) Treating 3 million by 2005: making it happen: the WHO strategy. 1–ed ed. Organization WH, editor Geneva, Switizerland: World Health Organization 61 p. http://www.who.int/3by5/publications/documents/en/Treating3millionby2005.pdf.

[5] Mocroft A, Reiss P, Kirk O, Mussini C, Girardi E, et al (2010) Is it safe to discontinue primary Pneumocystis jiroveci pneumonia prophylaxis in patients with virologically suppressed HIV infection and a CD4 cell count <200 cells/microL? Clin Infect Dis 51: 611–619. Available: http://www.ncbi.nlm.nih.gov/pubmed/20645862. 10.1086/655761 20645862

[6] Reynolds SJ, Sempa JB, Kiragga AN, Newell K, Nakigozi G, et al (2014) Is CD4 monitoring needed among ugandan clients achieving a virologic and immunologic response to treatment? AIDS Patient Care STDS 28: 575–578. Available: http://www.ncbi.nlm.nih.gov/pubmed/25290988. 10.1089/apc.2014.0086 25290988 PMC4227437

[7] Nakanjako D, Kiragga AN, Musick B, Yiannoutsos C, Wools-Kaloustian K, et al (2016) Frequency and impact of suboptimal immune recovery on first-line antiretroviral therapy (ART) within the IeDEA-East Africa cohort. AIDS. Available: http://www.ncbi.nlm.nih.gov/pubmed/26959510. 10.1097/QAD.0000000000001085 PMC5438045 26959510

[8] World Health Organization (2013) Consolidated Guidelines on the Use of Antiretroviral Drugs for Treatment and Preventing HIV Infection. Geneva, Switizerland http://apps.who.int/iris/bitstream/10665/85321/1/9789241505727_eng.pdf?ua=1. Accessed 22 March 2016.

[9] Sax PE (2013) Editorial Commentary : Can We Break the Habit of Routine CD4 Monitoring in HIV Care? Clin Infect Dis 56: 1344–1346. Available: http://cid.oxfordjournals.org/lookup/doi/10.1093/cid/cit008. 23315314 10.1093/cid/cit008

[10] Reynolds SJ, Nakigozi G, Newell K, Ndyanabo A, Galiwongo R, et al (2009) Failure of immunologic criteria to appropriately identify antiretroviral treatment failure in Uganda. AIDS 23: 697–700. Available: http://www.ncbi.nlm.nih.gov/pubmed/19209067. 10.1097/QAD.0b013e3283262a78 19209067 PMC2720562

[11] Kiragga AN, Castelnuovo B, Kamya MR, Moore R, Manabe YC (2012) Regional differences in predictive accuracy of WHO immunologic failure criteria. AIDS 26: 768–770. Available: http://www.ncbi.nlm.nih.gov/pubmed/22269974. 10.1097/QAD.0b013e32835143e3 22269974 PMC3812797

[12] Nash D, Katyal M, Brinkhof MWG, Keiser O, May M, et al (2008) Long-term immunologic response to antiretroviral therapy in low-income countries: a collaborative analysis of prospective studies. AIDS 22: 2291–2302. Available: http://www.ncbi.nlm.nih.gov/pubmed/18981768. 10.1097/QAD.0b013e3283121ca9 18981768 PMC2794130

[13] Sempa JB, Kiragga AN, Castelnuovo B, Kamya MR, Manabe YC (2013) Among patients with sustained viral suppression in a resource-limited setting, CD4 gains are continuous although gender-based differences occur. PLoS One 8: e73190 Available: http://www.scopus.com/inward/record.url?eid=2-s2.0-84883171455&partnerID=tZOtx3y1. Accessed 2 April 2015. 10.1371/journal.pone.0073190 24013838 PMC3754935

[14] Wandeler G, Gsponer T, Mulenga L, Garone D, Wood R, et al (2013) Zidovudine impairs immunological recovery on first-line antiretroviral therapy: collaborative analysis of cohort studies in southern Africa. AIDS 27: 2225–2232. Available: http://www.scopus.com/inward/record.url?eid=2-s2.0-84884502250&partnerID=tZOtx3y1. Accessed 2 April 2015. 10.1097/QAD.0b013e328362d887 23660577 PMC3815688

[15] Crawford KW, Wakabi S, Magala F, Kibuuka H, Liu M, et al (2015) Evaluation of treatment outcomes for patients on first-line regimens in US President’s Emergency Plan for AIDS Relief (PEPFAR) clinics in Uganda: predictors of virological and immunological response from RV288 analyses. HIV Med 16: 95–104. Available: http://www.scopus.com/inward/record.url?eid=2-s2.0-84921305133&partnerID=tZOtx3y1. Accessed 2 April 2015. 10.1111/hiv.12177 25124078

[16] Hermans SM, Kiragga AN, Schaefer P, Kambugu A, Hoepelman AIM, et al (2010) Incident tuberculosis during antiretroviral therapy contributes to suboptimal immune reconstitution in a large urban HIV clinic in sub-Saharan Africa. PLoS One 5: e10527 Available: http://www.scopus.com/inward/record.url?eid=2-s2.0-77956290926&partnerID=tZOtx3y1. Accessed 2 April 2015. 10.1371/journal.pone.0010527 20479873 PMC2866328

[17] Assefa A, Gelaw B, Getnet G, Yitayew G (2014) The effect of incident tuberculosis on immunological response of HIV patients on highly active anti-retroviral therapy at the university of Gondar hospital, northwest Ethiopia: a retrospective follow-up study. BMC Infect Dis 14: 468 Available: http://www.scopus.com/inward/record.url?eid=2-s2.0-84921637083&partnerID=tZOtx3y1. Accessed 2 April 2015. 10.1186/1471-2334-14-468 25164855 PMC4158052

[18] Moher D, Liberati A, Tetzlaff J, Altman DG, PRISMA Group (2009) Preferred reporting items for systematic reviews and meta-analyses: the PRISMA statement. BMJ 339: b2535 Available: http://www.ncbi.nlm.nih.gov/pubmed/19622551. 10.1136/bmj.b2535 19622551 PMC2714657

[19] Burnham JF (2006) Scopus database: a review. Biomed Digit Libr 3: 1 Available: http://www.ncbi.nlm.nih.gov/pubmed/16522216. 10.1186/1742-5581-3-1 16522216 PMC1420322

[20] Fellows I (2014) wordcloud: Word Clouds. http://cran.r-project.org/package=wordcloud.

[21] Peterson I, Togun O, de Silva T, Oko F, Rowland-Jones S, et al (2011) Mortality and immunovirological outcomes on antiretroviral therapy in HIV-1 and HIV-2-infected individuals in the Gambia. AIDS 25: 2167–2175. Available: http://www.scopus.com/inward/record.url?eid=2-s2.0-80055042755&partnerID=tZOtx3y1. Accessed 2 April 2015. 10.1097/QAD.0b013e32834c4adb 21881480

[22] Hawkins C, Chalamilla G, Okuma J, Spiegelman D, Hertzmark E, et al (2011) Sex differences in antiretroviral treatment outcomes among HIV-infected adults in an urban Tanzanian setting. AIDS 25: 1189–1197. Available: http://www.scopus.com/inward/record.url?eid=2-s2.0-79957640395&partnerID=tZOtx3y1. Accessed 2 April 2015. 10.1097/QAD.0b013e3283471deb 21505309

[23] Sudfeld CR, Isanaka S, Mugusi FM, Aboud S, Wang M, et al (2013) Weight change at 1 mo of antiretroviral therapy and its association with subsequent mortality, morbidity, and CD4 T cell reconstitution in a Tanzanian HIV-infected adult cohort. Am J Clin Nutr 97: 1278–1287. Available: http://www.scopus.com/inward/record.url?eid=2-s2.0-84878400920&partnerID=tZOtx3y1. Accessed 2 April 2015. 10.3945/ajcn.112.053728 23636235 PMC3652924

[24] Sudfeld CR, Wang M, Aboud S, Giovannucci EL, Mugusi FM, et al (2012) Vitamin D and HIV progression among Tanzanian adults initiating antiretroviral therapy. PLoS One 7: e40036 Available: http://www.scopus.com/inward/record.url?eid=2-s2.0-84863111779&partnerID=tZOtx3y1. Accessed 9 March 2015. 10.1371/journal.pone.0040036 22768212 PMC3386915

[25] Hardwick RJ, Amogne W, Mugusi S, Yimer G, Ngaimisi E, et al (2012) β-defensin genomic copy number is associated with HIV load and immune reconstitution in sub-saharan Africans. J Infect Dis 206: 1012–1019. Available: http://www.scopus.com/inward/record.url?eid=2-s2.0-84866101364&partnerID=tZOtx3y1. Accessed 2 April 2015. 10.1093/infdis/jis448 22837491

[26] Boullé C, Kouanfack C, Laborde-Balen G, Carrieri MP, Dontsop M, et al (2013) Task shifting HIV care in rural district hospitals in Cameroon: evidence of comparable antiretroviral treatment-related outcomes between nurses and physicians in the Stratall ANRS/ESTHER trial. J Acquir Immune Defic Syndr 62: 569–576. Available: http://www.scopus.com/inward/record.url?eid=2-s2.0-84876409785&partnerID=tZOtx3y1. Accessed 2 April 2015. 10.1097/QAI.0b013e318285f7b6 23337365

[27] Velen K, Lewis JJ, Charalambous S, Grant AD, Churchyard GJ, et al (2013) Comparison of tenofovir, zidovudine, or stavudine as part of first-line antiretroviral therapy in a resource-limited-setting: a cohort study. PLoS One 8: e64459 Available: http://www.scopus.com/inward/record.url?eid=2-s2.0-84877733753&partnerID=tZOtx3y1. Accessed 2 April 2015. 10.1371/journal.pone.0064459 23691224 PMC3653880

[28] Schomaker M, Egger M, Maskew M, Garone D, Prozesky H, et al (2013) Immune recovery after starting ART in HIV-infected patients presenting and not presenting with tuberculosis in South Africa. J Acquir Immune Defic Syndr 63: 142–145. Available: http://www.ncbi.nlm.nih.gov/pubmed/23364513. 10.1097/QAI.0b013e318288b39d 23364513 PMC3671097

[29] Hamers RL, Schuurman R, Sigaloff KCE, Wallis CL, Kityo C, et al (2012) Effect of pretreatment HIV-1 drug resistance on immunological, virological, and drug-resistance outcomes of first-line antiretroviral treatment in sub-Saharan Africa: a multicentre cohort study. Lancet Infect Dis 12: 307–317. Available: http://www.scopus.com/inward/record.url?eid=2-s2.0-84859002690&partnerID=tZOtx3y1. Accessed 24 March 2015. 10.1016/S1473-3099(11)70255-9 22036233

[30] Hamers RL, Zaaijer HL, Wallis CL, Siwale M, Ive P, et al (2013) HIV-HBV coinfection in Southern Africa and the effect of lamivudine- versus tenofovir-containing cART on HBV outcomes. J Acquir Immune Defic Syndr 64: 174–182. Available: http://www.scopus.com/inward/record.url?eid=2-s2.0-84885295369&partnerID=tZOtx3y1. Accessed 2 April 2015. 10.1097/QAI.0b013e3182a60f7d 23892239

[31] Maman D, Pujades-Rodriguez M, Subtil F, Pinoges L, McGuire M, et al (2012) Gender differences in immune reconstitution: a multicentric cohort analysis in sub-Saharan Africa. PLoS One 7: e31078 Available: http://www.scopus.com/inward/record.url?eid=2-s2.0-84857170838&partnerID=tZOtx3y1. Accessed 2 April 2015. 10.1371/journal.pone.0031078 22363550 PMC3281917

[32] Reda AA, Biadgilign S, Deribew A, Gebre B, Deribe K (2013) Predictors of change in CD4 lymphocyte count and weight among HIV infected patients on anti-retroviral treatment in Ethiopia: a retrospective longitudinal study. PLoS One 8: e58595 Available: http://www.scopus.com/inward/record.url?eid=2-s2.0-84875923724&partnerID=tZOtx3y1. Accessed 2 April 2015. 10.1371/journal.pone.0058595 23573191 PMC3616015

[33] Maskew M, MacPhail AP, Whitby D, Egger M, Fox MP (2013) Kaposi sarcoma-associated herpes virus and response to antiretroviral therapy: a prospective study of HIV-infected adults. J Acquir Immune Defic Syndr 63: 442–448. Available: http://www.scopus.com/inward/record.url?eid=2-s2.0-84880227939&partnerID=tZOtx3y1. Accessed 2 April 2015. 10.1097/QAI.0b013e3182969cc1 23614996 PMC3712196

[34] Mayanja BN, Shafer LA, Van der Paal L, Kyakuwa N, Ndembi N, et al (2012) Effect of pregnancy on immunological and virological outcomes of women on ART: a prospective cohort study in rural Uganda, 2004–2009. Trop Med Int Health 17: 343–352. Available: http://www.scopus.com/inward/record.url?eid=2-s2.0-84857361697&partnerID=tZOtx3y1. Accessed 2 April 2015. 10.1111/j.1365-3156.2011.02921.x 22212561

[35] Sarfo FS, Sarfo MA, Kasim A, Phillips R, Booth M, et al (2014) Long-term effectiveness of first-line non-nucleoside reverse transcriptase inhibitor (NNRTI)-based antiretroviral therapy in Ghana. J Antimicrob Chemother 69: 254–261. Available: http://www.scopus.com/inward/record.url?eid=2-s2.0-84890393346&partnerID=tZOtx3y1. Accessed 2 April 2015. 10.1093/jac/dkt336 24003181

[36] De Beaudrap P, Etard J-F, Diouf A, Ndiaye I, Guèye NF, et al (2009) Modeling CD4+ cell count increase over a six-year period in HIV-1-infected patients on highly active antiretroviral therapy in Senegal. Am J Trop Med Hyg 80: 1047–1053. Available: http://www.ncbi.nlm.nih.gov/pubmed/19478274. 19478274

[37] Vinikoor MJ, Joseph J, Mwale J, Marx MA, Goma FM, et al (2014) Age at antiretroviral therapy initiation predicts immune recovery, death, and loss to follow-up among HIV-infected adults in urban Zambia. AIDS Res Hum Retroviruses 30: 949–955. Available: http://www.scopus.com/inward/record.url?eid=2-s2.0-84907513063&partnerID=tZOtx3y1. Accessed 2 April 2015. 10.1089/AID.2014.0046 24998881 PMC4179921

[38] Kigozi BK, Sumba S, Mudyope P, Namuddu B, Kalyango J, et al (2009) The effect of AIDS defining conditions on immunological recovery among patients initiating antiretroviral therapy at Joint Clinical Research Centre, Uganda. AIDS Res Ther 6: 17 Available: http://www.scopus.com/inward/record.url?eid=2-s2.0-69349095853&partnerID=tZOtx3y1. Accessed 2 April 2015. 10.1186/1742-6405-6-17 19630949 PMC2731026

[39] Palladino C, Briz V, Bellón JM, Bártolo I, Carvalho P, et al (2013) Predictors of attrition and immunological failure in HIV-1 patients on highly active antiretroviral therapy from different healthcare settings in Mozambique. PLoS One 8: e82718 Available: http://www.scopus.com/inward/record.url?eid=2-s2.0-84893370669&partnerID=tZOtx3y1. Accessed 2 April 2015. 10.1371/journal.pone.0082718 24376569 PMC3869714

[40] Alemu Melsew Y (2013) Rate of Immunological Failure and its Predictors among Patients on Highly Active Antiretroviral Therapy at Debremarkos Hospital, Northwest Ethiopia: A Retrospective Follow up Study. J AIDS Clin Res 4 Available: http://www.scopus.com/inward/record.url?eid=2-s2.0-84879910986&partnerID=tZOtx3y1. Accessed 2 April 2015.

[41] Teshome W, Assefa A (2014) Predictors of immunological failure of antiretroviral therapy among HIV infected patients in Ethiopia: a matched case-control study. PLoS One 9: e115125 Available: http://www.scopus.com/inward/record.url?eid=2-s2.0-84919793261&partnerID=tZOtx3y1. Accessed 2 April 2015. 10.1371/journal.pone.0115125 25536416 PMC4275231

[42] Mudiope PK, Kim S, Wabwire D, Nyende L, Bagenda D, et al (2013) Long-term clinical and immunologic outcomes of HIV-infected women with and without previous exposure to nevirapine. Trop Med Int Health 18: 344–351. Available: http://www.scopus.com/inward/record.url?eid=2-s2.0-84873987379&partnerID=tZOtx3y1. Accessed 2 April 2015. 10.1111/tmi.12054 23289497 PMC6746559

[43] Anude CJ, Eze E, Onyegbutulem HC, Charurat M, Etiebet M-A, et al (2013) Immuno-virologic outcomes and immuno-virologic discordance among adults alive and on anti-retroviral therapy at 12 months in Nigeria. BMC Infect Dis 13: 113 Available: http://www.scopus.com/inward/record.url?eid=2-s2.0-84874397717&partnerID=tZOtx3y1. Accessed 2 April 2015. 10.1186/1471-2334-13-113 23452915 PMC3599241

[44] Diabaté S, Alary M (2009) Criteria for initiating highly active antiretroviral therapy and short-term immune response among HIV-1-infected patients in Côte d’Ivoire. HIV Med 10: 640–646. Available: http://www.scopus.com/inward/record.url?eid=2-s2.0-70449336265&partnerID=tZOtx3y1. Accessed 2 April 2015. 10.1111/j.1468-1293.2009.00736.x 19659945

[45] Nglazi MD, Lawn SD, Kaplan R, Kranzer K, Orrell C, et al (2011) Changes in programmatic outcomes during 7 years of scale-up at a community-based antiretroviral treatment service in South Africa. J Acquir Immune Defic Syndr 56: e1–8. Available: http://www.scopus.com/inward/record.url?eid=2-s2.0-78650688259&partnerID=tZOtx3y1. Accessed 2 April 2015. 10.1097/QAI.0b013e3181ff0bdc 21084996 PMC3776048

[46] Efraim L, Peck RN, Kalluvya SE, Kabangila R, Mazigo HD, et al (2013) Schistosomiasis and impaired response to antiretroviral therapy among HIV-infected patients in Tanzania. J Acquir Immune Defic Syndr 62: e153–6. Available: http://www.scopus.com/inward/record.url?eid=2-s2.0-84876350380&partnerID=tZOtx3y1. Accessed 2 April 2015. 10.1097/QAI.0b013e318282a1a4 23760064 PMC3682228

[47] Alemu H, Haile Mariam D, Tsui A, Ahmed S, Shewamare A (2012) Effect of depressive symptoms and social support on weight and CD4 count increase at HIV clinic in Ethiopia. AIDS Care 24: 866–876. Available: http://www.scopus.com/inward/record.url?eid=2-s2.0-84863643210&partnerID=tZOtx3y1. Accessed 2 April 2015. 10.1080/09540121.2011.648160 22273149

[48] McKinnon LR, Kimani M, Wachihi C, Nagelkerke NJ, Muriuki FK, et al (2010) Effect of baseline HIV disease parameters on CD4+ T cell recovery after antiretroviral therapy initiation in Kenyan women. PLoS One 5: e11434 Available: http://www.scopus.com/inward/record.url?eid=2-s2.0-77955393670&partnerID=tZOtx3y1. Accessed 2 April 2015. 10.1371/journal.pone.0011434 20625393 PMC2896395

[49] Maskew M, Fox MP, van Cutsem G, Chu K, Macphail P, et al (2013) Treatment response and mortality among patients starting antiretroviral therapy with and without Kaposi sarcoma: a cohort study. PLoS One 8: e64392 Available: http://www.scopus.com/inward/record.url?eid=2-s2.0-84878758600&partnerID=tZOtx3y1. Accessed 2 April 2015. 10.1371/journal.pone.0064392 23755122 PMC3673971

[50] Takuva S, Westreich D, Menezes CN, McNamara L, Sanne I, et al (2012) Antiretroviral therapy initiation during tuberculosis treatment and HIV-RNA and CD4 T-lymphocyte responses. Int J Tuberc Lung Dis 16: 1358–1364. Available: http://www.scopus.com/inward/record.url?eid=2-s2.0-84866443079&partnerID=tZOtx3y1. Accessed 2 April 2015. 10.5588/ijtld.11.0769 22863288 PMC3727657

[51] Giacometti A, Butini L, Cirioni O, Costantini A, Montroni M, et al (2010) [Durability and tolerability of long-term nevirapine-based HAART]. Infez Med 18: 20–26. Available: http://www.ncbi.nlm.nih.gov/pubmed/20424522. 20424522

[52] Boulle A, Orrel C, Kaplan R, Van Cutsem G, McNally M, et al (2007) Substitutions due to antiretroviral toxicity or contraindication in the first 3 years of antiretroviral therapy in a large South African cohort. Antivir Ther 12: 753–760. Available: http://www.ncbi.nlm.nih.gov/pubmed/17713158. 17713158 10.1177/135965350701200508

[53] Rizopoulos D (2012) Joint models for longitudinal and time-to-event data—Index. Most. pp. 55–56. papers3://publication/uuid/3CD351D4-BD94-483E-87CB-D95574B1A92A.

[54] Pandeya N (2005) Repeated Occurrence of Basal Cell Carcinoma of the Skin and Multifailure Survival Analysis: Follow-up Data from the Nambour Skin Cancer Prevention Trial. Am J Epidemiol 161: 748–754. Available: http://aje.oupjournals.org/cgi/doi/10.1093/aje/kwi098. 15800267 10.1093/aje/kwi098

[55] Therneau TM, Grambsch Patricia M. (2000) Modeling Survival Data: Extending the Cox Model. Newyork: Springer http://www.escarela.com/archivo/anahuac/03o/residuals.pdf.

[56] Harrell FE (2001) Regression Modeling Strategies. 1ed ed. Bickel P, Diggle P, Fienberg S, Krickeberg K, Olkin I, et al, editors New York, NY: Springer New York 560 p. http://link.springer.com/10.1007/978-1-4757-3462-1.

[57] Fitzmaurice G, Davidian M, Verbeke G, Molenberghs G (2009) Longitudinal data analysis. Fitzmaurice, Garrett (Boston M, Davidian, Marie (Raleigh NC, Verbeke, Geert (Leuven B, Molenberghs, Geert (Diepenbeek B, editors Chapman and Hall/CRC. 633 p.

[58] Gordon CL, Cheng AC, Cameron PU, Bailey M, Crowe SM, et al (2015) Quantitative Assessment of Intra-Patient Variation in CD4+ T Cell Counts in Stable, Virologically-Suppressed, HIV-Infected Subjects. PLoS One 10: e0125248 Available: http://www.ncbi.nlm.nih.gov/pubmed/26110761. 10.1371/journal.pone.0125248 26110761 PMC4482322

[59] Rood Y, Goulmy E, Blokland E, Pool J, Rood J, et al (2008) Month-related variability in immunological test results; implications for immunological follow-up studies. Clin Exp Immunol 86: 349–354. Available: http://doi.wiley.com/10.1111/j.1365-2249.1991.tb05821.x. 10.1111/j.1365-2249.1991.tb05821.x PMC1554130 1834381

[60] Raboud JM, Haley L, Montaner JS, Murphy C, Januszewska M, et al (1995) Quantification of the variation due to laboratory and physiologic sources in CD4 lymphocyte counts of clinically stable HIV-infected individuals. J Acquir Immune Defic Syndr Hum Retrovirol 10 Suppl 2: S67–73. Available: http://www.ncbi.nlm.nih.gov/pubmed/7552516. 7552516

[61] Corbeau P, Reynes J (2011) Immune reconstitution under antiretroviral therapy: The new challenge in HIV-1 infection. Blood 117: 5582–5590. 10.1182/blood-2010-12-322453 21403129

[62] Fung IC-H, Gambhir M, van Sighem A, de Wolf F, Garnett GP (2012) The Clinical Interpretation of Viral Blips in HIV Patients Receiving Antiviral Treatment. JAIDS J Acquir Immune Defic Syndr 60: 5–11. 10.1097/QAI.0b013e3182487a20 22267019

[63] Greenland S (1990) Randomization, statistics, and causal inference. Epidemiology 1: 421–429. Available: http://www.ncbi.nlm.nih.gov/pubmed/2090279. 2090279 10.1097/00001648-199011000-00003

[64] Poole C (2001) Low P-values or narrow confidence intervals: which are more durable? Epidemiology 12: 291–294. Available: http://www.ncbi.nlm.nih.gov/pubmed/11337599. 11337599 10.1097/00001648-200105000-00005

[65] Greenland S, Pearl J, Robins JM (1999) Causal diagrams for epidemiologic research. Epidemiology 10: 37–48. Available: http://www.ncbi.nlm.nih.gov/pubmed/9888278. 9888278

[66] Hoeting JA, Madigan DM, Raftery AE, Volinsky CT (1999) Bayesian model averaging: A tutorial. Stat Sci 14: 382–417.

[67] Becher H (1992) The concept of residual confounding in regression models and some applications. Stat Med 11: 1747–1758. Available: http://www.ncbi.nlm.nih.gov/pubmed/1485057. 1485057 10.1002/sim.4780111308

[68] Altman DG, Lausen B, Sauerbrei W, Schumacher M (1994) Dangers of using “optimal” cutpoints in the evaluation of prognostic factors. J Natl Cancer Inst 86: 829–835. Available: http://www.ncbi.nlm.nih.gov/pubmed/8182763. 8182763 10.1093/jnci/86.11.829

[69] Lesaffre E, Lawson AB (2012) Bayesian Biostatistics. Chichester, UK: John Wiley & Sons, Ltd http://doi.wiley.com/10.1002/9781119942412.

[70] Nakashima H, Yamamoto N, Masuda M, Fujii N (1993) Defensins inhibit HIV replication in vitro. AIDS 7: 1129 Available: http://www.ncbi.nlm.nih.gov/pubmed/8397954. 8397954 10.1097/00002030-199308000-00019

[71] Stone SF, Price P, French MA (2005) Dysregulation of CD28 and CTLA-4 expression by CD4 T cells from previously immunodeficient HIV-infected patients with sustained virological responses to highly active antiretroviral therapy. HIV Med 6: 278–283. Available: http://www.ncbi.nlm.nih.gov/pubmed/16011533. 10.1111/j.1468-1293.2005.00307.x 16011533

