# Supplementary material for: Systematic review of statistically-derived models of immunological response in HIV-infected adults on antiretroviral therapy in Sub-Saharan Africa
Source: PLoS One. 2017 Feb 15;12(2):e0171658. doi: 10.1371/journal.pone.0171658 (PMC5310790; doi:10.1371/journal.pone.0171658)
Supplement: S2 File — (DOCX) [file pone.0171658.s002.docx]

**Appendix 1:** SCOPUS search strategy

*Immune response: 402,690*

[TITLE-ABS-KEY ( **"Immune response"**  OR  **"CD4 count change"**  OR  **"CD4 trajectory"**  OR  **"CD4 count"**  OR  **"CD4"**  OR  **"cd4 lymphocyte count"**  OR  **"CD4 counts"**  OR  **"immunity cellular"**  OR  **"cellular immune response"**  OR  **"cellular immune recovery"**  OR  **"cellular immune reconstitution"**  OR  **"cellular immune trajectory"**  OR  **"CD4 recovery"**  OR  **"CD4 trajectory"**  OR  **"CD4 response"**  OR  **"CD4 gains"** )  AND  ( LIMIT-TO ( LANGUAGE ,  **"English"** ) )](http://www-scopus-com.ez.sun.ac.za/search/save/action.url?activity=allAction&userSearchID=12&origin=savedsearch)

*HAART: 54,159*

[TITLE-ABS-KEY ( **"antiretroviral therapy"**  OR  **"HAART"**  OR  **"combination antiretroviral therapy"**  OR  **"cART"**  OR  **"highly active antiretroviral therapy"** )  AND  ( LIMIT-TO ( LANGUAGE , **"English"** ) )](http://www-scopus-com.ez.sun.ac.za/search/save/action.url?activity=allAction&userSearchID=5&origin=savedsearch)

*Statistical Model: 1,022,199*

[TITLE-ABS-KEY ( **"Likelihood Functions"**  OR  **"Linear Models"**  OR  **"Logistic Models"**  OR **"Proportional Hazards Models"**  OR  **"Least-Squares Analysis"**  OR  **"Nomograms"**  OR **"Models, Statistical"**  OR  **"regression"** )  AND  ( LIMIT-TO ( LANGUAGE ,  **"English"** ) )](http://www-scopus-com.ez.sun.ac.za/search/save/action.url?activity=allAction&userSearchID=7&origin=savedsearch)

*SSA: 555,920*

[TITLE-ABS-KEY ( **"Angola"**  OR  **"Benin"**  OR  **"Botswana"**  OR  **"Burkina Faso"**  OR  **"Burundi"** OR  **"Cameroon"**  OR  **"Cape Verde"**  OR  **"Central African Republic"**  OR  **"Chad"**  OR **"Comoros"**  OR  **"Congo"**  OR  **"Côte d'Ivoire"**  OR  **"Djibouti"**  OR  **"Equatorial Guinea"**  OR **"Eritrea"**  OR  **"Ethiopia"**  OR  **"Gabon"**  OR  **"Gambia"**  OR  **"Ghana"**  OR  **"Guinea"**  OR **"Guinea-Bissau"**  OR  **"Kenya"**  OR  **"Lesotho"**  OR  **"Liberia"**  OR  **"Madagascar"**  OR **"Malawi"**  OR  **"Mali"**  OR  **"Mauritania"**  OR  **"Mauritius"**  OR  **"Mozambique"**  OR  **"Namibia"** OR  **"Niger"**  OR  **"Nigeria"**  OR  **"Réunion"**  OR  **"Rwanda"**  OR  **"Sao Tome and Principe"**  OR **"Senegal"**  OR  **"Seychelles"**  OR  **"Sierra Leone"**  OR  **"Somalia"**  OR  **"South Africa"**  OR **"Sudan"**  OR  **"Swaziland"**  OR  **"Tanzania"**  OR  **"Togo"**  OR  **"Uganda"** OR  **"Zambia"**  OR  **"Zimbabwe"**  OR  **"Sub-Saharan Africa"**  OR  **"Subsaharan Africa"**  OR  **"Africa, Sub-Saharan"**  OR  **"south of sahara"** )  AND  ( LIMIT-TO ( LANGUAGE , **"English"** ) )](http://www-scopus-com.ez.sun.ac.za/search/save/action.url?activity=allAction&userSearchID=16&origin=savedsearch)

*Immune response AND HAART = 20,476*

[( ( TITLE-ABS-KEY ( **"Immune response"**  OR  **"CD4 count change"**  OR  **"CD4 trajectory"**  OR  **"CD4 count"**  OR  **"CD4"**  OR  **"cd4 lymphocyte count"** OR  **"CD4 counts"**  OR  **"immunity cellular"**  OR  **"cellular immune response"**  OR  **"cellular immune recovery"**  OR  **"cellular immune reconstitution"**  OR  **"cellular immune trajectory"** OR  **"CD4 recovery"**  OR  **"CD4 trajectory"**  OR  **"CD4 response"**  OR  **"CD4 gains"** ) )  AND ( TITLE-ABS-KEY ( **"antiretroviral therapy"**  OR  **"HAART"**  OR  **"combination antiretroviral therapy"**  OR  **"cART"**  OR  **"highly active antiretroviral therapy"** ) ) )  AND (LIMIT-TO (LANGUAGE , **"English"** ) )](http://www-scopus-com.ez.sun.ac.za/search/save/action.url?activity=allAction&userSearchID=13&origin=savedsearch)

*(Immune response AND HAART)* ***AND*** *SSA =2588*

[( ( TITLE-ABS-KEY ( **"Immune response"**  OR  **"CD4 count change"**  OR  **"CD4 trajectory"**  OR  **"CD4 count"**  OR  **"CD4"**  OR  **"cd4 lymphocyte count"** OR  **"CD4 counts"**  OR  **"immunity cellular"**  OR  **"cellular immune response"**  OR  **"cellular immune recovery"**  OR  **"cellular immune reconstitution"**  OR  **"cellular immune trajectory"** OR  **"CD4 recovery"**  OR  **"CD4 trajectory"**  OR  **"CD4 response"**  OR  **"CD4 gains"** ) )  AND ( TITLE-ABS-KEY ( **"antiretroviral therapy"**  OR  **"HAART"**  OR  **"combination antiretroviral therapy"**  OR  **"cART"**  OR  **"highly active antiretroviral therapy"** ) ) ) )  AND  ( TITLE-ABS-KEY ( **"Angola"**  OR  **"Benin"**  OR  **"Botswana"**  OR  **"Burkina Faso"**  OR  **"Burundi"**  OR **"Cameroon"**  OR  **"Cape Verde"**  OR  **"Central African Republic"**  OR  **"Chad"**  OR **"Comoros"**  OR  **"Congo"**  OR  **"Côte d'Ivoire"**  OR  **"Djibouti"**  OR  **"Equatorial Guinea"**  OR **"Eritrea"**  OR  **"Ethiopia"**  OR  **"Gabon"**  OR  **"Gambia"**  OR  **"Ghana"**  OR  **"Guinea"**  OR **"Guinea-Bissau"**  OR  **"Kenya"**  OR  **"Lesotho"**  OR  **"Liberia"**  OR  **"Madagascar"**  OR **"Malawi"**  OR  **"Mali"**  OR  **"Mauritania"**  OR  **"Mauritius"**  OR  **"Mozambique"**  OR  **"Namibia"** OR  **"Niger"**  OR  **"Nigeria"**  OR  **"Réunion"**  OR  **"Rwanda"**  OR  **"Sao Tome and Principe"**  OR **"Senegal"**  OR  **"Seychelles"**  OR  **"Sierra Leone"**  OR  **"Somalia"**  OR  **"South Africa"**  OR **"Sudan"**  OR  **"Swaziland"**  OR  **"Tanzania"**  OR  **"Togo"**  OR  **"Uganda"**  OR  **"Zambia"**  OR  **"Zimbabwe"**  OR  **"Sub-Saharan Africa"**  OR  **"Subsaharan Africa"**  OR  **"Africa, Sub-Saharan"**  OR  **"south of sahara"** ) ) )  AND  ( LIMIT-TO ( LANGUAGE ,  **"English"** ) )](http://www-scopus-com.ez.sun.ac.za/search/save/action.url?activity=allAction&userSearchID=17&origin=savedsearch)

*(Immune response AND HAART AND SSA) AND Statistical models = 614*

[( ( TITLE-ABS-KEY ( **"Immune response"**  OR  **"CD4 count change"**  OR  **"CD4 trajectory"**  OR  **"CD4 count"**  OR  **"CD4"**  OR  **"cd4 lymphocyte count"** OR  **"CD4 counts"**  OR  **"immunity cellular"**  OR  **"cellular immune response"**  OR  **"cellular immune recovery"**  OR  **"cellular immune reconstitution"**  OR  **"cellular immune trajectory"** OR  **"CD4 recovery"**  OR  **"CD4 trajectory"**  OR  **"CD4 response"**  OR  **"CD4 gains"** ) )  AND ( TITLE-ABS-KEY ( **"antiretroviral therapy"**  OR  **"HAART"**  OR  **"combination antiretroviral therapy"**  OR  **"cART"**  OR  **"highly active antiretroviral therapy"** ) ) ) )  AND  ( TITLE-ABS-KEY ( **"Angola"**  OR  **"Benin"**  OR  **"Botswana"**  OR  **"Burkina Faso"**  OR  **"Burundi"**  OR **"Cameroon"**  OR  **"Cape Verde"**  OR  **"Central African Republic"**  OR  **"Chad"**  OR **"Comoros"**  OR  **"Congo"**  OR  **"Côte d'Ivoire"**  OR  **"Djibouti"**  OR  **"Equatorial Guinea"**  OR **"Eritrea"**  OR  **"Ethiopia"**  OR  **"Gabon"**  OR  **"Gambia"**  OR  **"Ghana"**  OR  **"Guinea"**  OR **"Guinea-Bissau"**  OR  **"Kenya"**  OR  **"Lesotho"**  OR  **"Liberia"**  OR  **"Madagascar"**  OR **"Malawi"**  OR  **"Mali"**  OR  **"Mauritania"**  OR  **"Mauritius"**  OR  **"Mozambique"**  OR  **"Namibia"** OR  **"Niger"**  OR  **"Nigeria"**  OR  **"Réunion"**  OR  **"Rwanda"**  OR  **"Sao Tome and Principe"**  OR **"Senegal"**  OR  **"Seychelles"**  OR  **"Sierra Leone"**  OR  **"Somalia"**  OR  **"South Africa"**  OR **"Sudan"**  OR  **"Swaziland"**  OR  **"Tanzania"**  OR  **"Togo"**  OR  **"Uganda"** OR  **"Zambia"**  OR  **"Zimbabwe"**  OR  **"Sub-Saharan Africa"**  OR  **"Subsaharan Africa"**  OR  **"Africa, Sub-Saharan"**  OR  **"south of sahara"** ) ) )  AND  ( TITLE-ABS-KEY ( **"Likelihood Functions"**  OR  **"Linear Models"**  OR  **"Logistic Models"**  OR **"Proportional Hazards Models"**  OR  **"Least-Squares Analysis"**  OR  **"Nomograms"**  OR **"Models, Statistical"**  OR  **"regression"** ) )  AND  ( LIMIT-TO ( LANGUAGE ,  **"English"** ) )](http://www-scopus-com.ez.sun.ac.za/search/save/action.url?activity=allAction&userSearchID=17&origin=savedsearch)
